# Estimating Drug Efficacy with a Diet-Induced NASH Model in Chimeric Mice with Humanized Livers

**DOI:** 10.3390/biomedicines9111647

**Published:** 2021-11-09

**Authors:** Keishi Kisoh, Go Sugahara, Yuko Ogawa, Suzue Furukawa, Yuji Ishida, Takeshi Okanoue, Michinori Kohara, Chise Tateno

**Affiliations:** 1 Research and Development Department, PhoenixBio Co., Ltd., 3-4-1 Kagamiyama, Higashihiroshima 739-0046, Japan; keishi.kisoh@phoenixbio.co.jp (K.K.); gou.sugahara@phoenixbio.co.jp (G.S.); yogawa@phoenixbio.co.jp (Y.O.); sfurukawa@phoenixbio.co.jp (S.F.); yuji.ishida@phoenixbio.co.jp (Y.I.); 2 Research Center for Hepatology and Gastroenterology, Hiroshima University, 1-2-3 Kasumi, Minami-ku, Hiroshima 734-8551, Japan; 3 Department of Gastroenterology and Hepatology, Saiseikai Suita Hospital, 1-2 Kawazonocho, Suita 564-0013, Japan; okanoue@suita.saiseikai.or.jp; 4 Department of Microbiology and Cell Biology, Tokyo Metropolitan Institute of Medical Science, 2-1-6 Kamikitazawa, Setagaya-ku, Tokyo 156-8506, Japan; kohara-mc@igakuken.or.jp

**Keywords:** human liver chimeric mice, NAFLD/NASH, ballooning hepatocytes, Mallory–Denk body, human disease animal model

## Abstract

Nonalcoholic fatty liver disease/steatohepatitis (NAFLD/NASH) is the most common liver disorder in developed countries. Although many new therapeutics for NASH are present in the drug development pipeline, there are still no approved drugs. One of the reasons that makes NASH drug development challenging is the lack of appropriate animal NASH models that resolve issues arising from inter-species differences between humans and rodents. In the present study, we developed a choline-deficient, L-amino-acid-defined, high-fat-diet (CDAHFD)-induced human NASH model using human liver chimeric mice. We demonstrated human hepatocyte injury by an elevation of plasma human alanine aminotransferase 1 in mice fed CDAHFD. Histological analysis showed that CDAHFD feeding induced similar histological changes to human NASH patients, including ballooning, inflammation, apoptosis, regeneration of human hepatocytes, and pericellular and perisinusoidal fibrosis. The chimeric mice fed CDAHFD were treated with a peroxisome-proliferator-activated receptor α/δ agonist, Elafibranor. Elafibranor ameliorated steatosis, ballooning of hepatocytes, and preserved fibrosis progression. We developed a novel humanized NASH model that can elucidate pathophysiological mechanisms and predict therapeutic efficacy in human NASH. This model will be useful in exploring new drugs and biomarkers in the early stages of human NASH.

## 1. Introduction

Nonalcoholic fatty liver disease (NAFLD) is now recognized as the most common liver disease in developed countries. One-fourth of the adult population worldwide suffers from NAFLD [1]. NAFLD is recognized as a hepatic phenotype of metabolic syndrome and is strongly associated with obesity, insulin resistance, and hyperlipidemia [2,3,4]. It is predicted that the number of NAFLD patients will continue to increase greatly.

NAFLD can exhibit a large spectrum of pathological changes—from simple steatosis to severe liver injury. NAFLD patients often develop nonalcoholic steatohepatitis (NASH), the progressive form of NAFLD, which is characterized by inflammation, hepatocyte degeneration-like ballooning, and fibrosis. This may further lead to cirrhosis and hepatocellular carcinoma [5,6]. However, the pathological mechanism of NAFLD is still not fully understood due to limitations in the timing, frequency, and volume of tissue sampling from patients.

Genetic and/or dietary animal models have been used in non-clinical trials and basic studies. Among them, diet-induced NASH models are thought to reflect clinical disease progression [5,7,8]. Recently, a choline-deficient, L-amino-acid-defined, high-fat diet (CDAHFD) has been used in many NASH studies [9,10]. CDAHFD was formulated to include a small amount of methionine (0.1%) and 60% of kcal from fat [11]. The deficiency of choline and methionine, which are lipotropic factors, causes impaired β-oxidation and the production of very-low-density lipoproteins from triglycerides [12]. As a result, these impairments induce fat accumulation in the liver, resulting in inflammation and fibrosis. CDAHFD feeding has also been reported to induce cirrhosis and/or hepatocellular carcinoma after long-term feeding [13]. Although this pathology in mice resembles the progression of human NASH, it does not fully mimic the metabolic changes.

We developed chimeric mice with humanized livers by the transplantation of human hepatocytes (h-heps) into urokinase-type plasminogen activator cDNA/severe combined immunodeficient (cDNA-uPA/SCID) mice [14]. The host mouse liver was replaced with h-heps, which showed a human gene expression profile, including phase I and II metabolic enzymes and transporters [15,16]. These mice have been used for drug efficacy studies and the prediction of drug metabolism and pharmacokinetics in humans [17]. Hence, it is thought that chimeric mice with human livers are useful for developing new drugs for human liver diseases, especially whose target genes or proteins are expressed in human hepatocytes. Furthermore, our previous study demonstrated that the livers of chimeric mice developed steatosis due to human growth hormone (h-GH) deficiency [18] but did not progress to liver injury and subsequent fibrosis. Therefore, it is also useful for elucidating the pathological mechanism of the progression from simple steatosis to NASH.

Recently, drug candidates targeting nuclear receptors, including PPARs, have been developed. PPAR is expressed in hepatocytes and plays an important role in regulating biological processes such as metabolism, inflammation, and differentiation [19,20,21,22]. There are species differences in PPARs between humans and rodents. For example, the expression of hepatic PPARα is lower in humans than in mice [23,24], and PPARα-regulated genes differ between human and mouse hepatocytes [25]. We previously demonstrated human and mouse species differences of the PPARα agonist fenofibrate, on peroxisome proliferation using chimeric mice with human hepatocytes [26,27]. To resolve issues arising from inter-species differences between humans and rodents, the chimeric mice with humanized livers will be essential to elucidate the efficacy and toxicity of drug candidates targeting human genes or proteins.

Elafibranor (GFT505), a selective dual peroxisome proliferator-activated receptor (PPAR) α/δ agonist, has been shown to ameliorate steatosis, inflammation, and fibrosis without adverse side effects in animal models and clinical trials [28,29,30,31]. Additionally, Elafibranor also improved abnormalities in glucose and lipid homeostasis and insulin resistance, which are closely linked to NASH pathogenesis [32,33].

In the present study, we aimed to develop a NASH murine model by using CDAHFD-fed human liver chimeric mice. In addition, we investigated whether this model is useful for the preclinical study of NASH drug development using Elafibranor targeting human PPARα/δ.

## 2. Materials and Methods

### 2.1. Animal and Generation of a CDAHFD-Induced NASH Model

The chimeric mice with h-heps were generated as previously described [14]. Cryopreserved h-heps (Lot; BD195: 2-year-old, Hispanic female, 1 × 10^6^ hepatocytes, BD Biosciences, Ann Arbor, MI, USA) were transplanted into 2- to 4-week-old hemizygous cDNA-uPA/SCID mice through the spleen under anesthesia. Male human liver chimeric mice (15 to 18-weeks of age, PXB-mice^®^) with a replacement index greater than 90%, calculated by the blood h-Alb concentration [14], were used for this study. These mice were divided into two groups categorized by body weight and blood h-Alb concentration (*n* = 4 per group). The mice were fed a CDAHFD consisting of 60 kcal% fat and 0.1% methionine by weight (A06071302, Research Diets Inc, NewBrunswick, NJ, USA) or a standard diet (CRF-1, Oriental yeast Co., Ltd., Tokyo, Japan) for 8 or 12 weeks. All experimental animals were housed with environmental enrichments under pathogen-free conditions and maintained in a 12 h light/dark cycle with sterilized water and diet available ad libitum.

### 2.2. Elafibranor Efficacy Study for the NASH Model

Elafibranor (Sunshine Chem Co., Ltd., Shanghai, China) was incorporated into CDAHFD (estimated dose of 30 mg/kg/day). The efficacy of Elafibranor was investigated in two study designs, which are preservation and therapeutic studies. In the former, normal chimeric mice with human hepatocytes were fed a CDAHFD or Elafibranor-containing CDAHFD for 8 weeks. In the latter, the human chimeric mice were fed a CDAHFD for 8 weeks and then changed to an Elafibranor-containing CDAHFD or continued with a CDAHFD for 6 weeks.

### 2.3. Measurement of Biochemical Markers

Blood samples were collected every 2 weeks, and then plasma or serum was extracted. ALT activity was measured by Fuji DRI-CHEM (Fuji FILM, Tokyo, Japan). Blood h-Alb levels were measured by immunonephelometry in a JEOL BM6050 autoanalyzer (JEOL, Tokyo, Japan) using LZ Reagent Eiken Alb II (Eiken Chemical, Tokyo, Japan). The plasma or serum levels of h-ALT1 and mouse M2BP level were measured according to the manufacturer’s protocols by using h-ALT1 ELISA kit (PhoenixBio, Co., Ltd., Higashihiroshima, Japan) [34] and Mac-2 Binding Protein assay kit (IBL Co., LTD., Fujioka, Japan), respectively.

### 2.4. Histological Analysis

Formalin-fixed, four micrometers thick, paraffin sections of mouse liver were prepared for hematoxylin and eosin (H&E) staining, immunohistochemistry, Sirius Red staining, or TUNEL staining. For immunohistochemistry, sections were incubated with the primary antibody at 4 °C after blocking, and then were re-incubated with the corresponding secondary antibody at room temperature. Primary antibodies for immunohistochemical analysis used in this study are shown in Supplementary Table S1: the replacement index of h-heps was calculated on STEM 121 immunostaining sections from two liver lobes as the ratio of the STEM 121-positive area to the entire area. Sirius Red staining was performed using Picrosirius Red Stain Kit (Polysciences, Inc., Warrington, PA, USA). Paraffin-embedded liver tissues were subjected to TUNEL analysis using an ApopTag Peroxidase In Situ Apoptosis Detection Kit (Chemicon International, Temecula, CA, USA). The nucleus was stained with hematoxylin as a counterstain. Seven-micrometer-thick frozen sections of mouse liver were prepared for Oil Red O staining. The sections were fixed with 10% formalin for 10 min and then stained with Oil Red O solution (Muto pure chemical Co., Ltd., Tokyo, Japan). All images were acquired by the BZ-X710 microscope (Keyence, Osaka, Japan). Quantification of the positively stained area and cells was calculated using BZ-X analysis software (Keyence).

### 2.5. Real-Time Quantitative Reverse Transcriptase-PCR (qRT-PCR)

Total RNA was isolated from each liver sample using RNeasy Mini Kit (QIAGEN N.V., Venlo, Netherlands). cDNA was synthesized according to manufacturer’s protocols using 1 μg of RNA, SuperScript Ⅲ reverse transcriptase and oligo-dT primers (Life Technologies, Carlsbad, CA, USA). Real-time qRT-PCR for mRNA expression was performed using SYBR Green PCR Master Mix on ABI 7500 Real-Time PCR System (Applied Biosystems, Tokyo, Japan). Experimental conditions were standardized to achieve an initial denaturation step at 95 °C for 10 min, followed by 40 cycles at 95 °C for 15 s, and 60 °C for 1 min. The sequence of each primer is shown in Supplementary Table S2. We ensured that mouse or human primers were specific for each species and did not cross-react with each other. The human gene expression level was normalized against a human glyceraldehyde-3-phosphate dehydrogenase (h-GAPDH) gene.

### 2.6. Measurement of Liver Oxidative Stress

Glutathione disulfide (GSSG) and reduced glutathione (GSH) levels in the liver were measured by using GSSG/GSH Quantification Kit (Dojindo Laboratories, Kamimashiki, Japan). Frozen liver samples were homogenized in 5% 5-Sulfosalicylic acid solution using glass–teflon homogenizer. After, the supernatant was collected after centrifugation (8000× *g*, 10 min, 4 °C) and was used in the assay. SOD activity in the liver was measured using SOD assay kit-WST (Dojindo Laboratories). Frozen liver samples were homogenized in sucrose buffer (0.25 M sucrose, 10 mM Tris-HCl pH 7.4, and 1 mM EDTA) using glass–teflon homogenizer. After centrifugation (10,000× *g*, 60 min, 4 °C), the supernatant was collected and measured SOD activity. These measurements were performed according to the manufacturer’s protocols.

### 2.7. Statistical Analysis

The results were expressed as the mean ± standard deviation. Statistical analyses were performed using Statcel 4 software. Differences between the two groups were evaluated using an unpaired *t* test. Statistical comparison among multiple groups was evaluated using an analysis of variance (ANOVA), followed by Tukey’s post hoc test or repeated measure ANOVA. *p* values less than 0.05 were considered significant.

## 3. Results

### 3.1. CDAHFD Feeding Decreased Body and Liver Weights and Deteriorated Liver Function of Chimeric Mice with H-Heps

**Figure 1:**
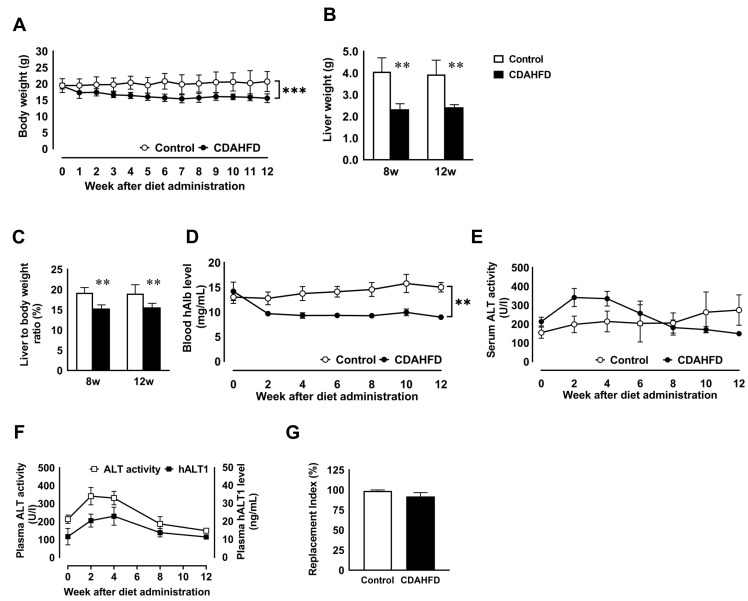
Changes in the physical and biochemical profiles of human chimeric mice after CDAHFD feeding. Time-course changes in the body weight of the CDAHFD (closed circle) or control (open circle) groups (**A**). The liver weight (**B**) and ratio of liver weight to body weight (**C**) of the control and CDAHFD-fed mice at week 8 or 12. Open and closed bars show the control and CDAHFD-fed mice, respectively. Time-course changes in the level of blood human albumin (**D**) and plasma ALT activity (**E**) of the CDAHFD or control group. The time-course changes in plasma ALT activity and the human-specific ALT1 concentration in the plasma of the CDAHFD group (**F**). Replacement index of the h-heps in chimeric mouse livers (**G**). Results are represented as the mean ± standard deviation (*n* = 3 or 4 per group). ** Significant difference from the control group (*p* < 0.01). *** Significant difference from the control group (*p* < 0.001). CDAHFD: choline-deficient, L-amino-acid-defined, high-fat diet, ALT: alanine aminotransferase.

Chimeric mice with h-heps were fed a standard diet or CDAHFD for 12 weeks. We first determined the changes in body weight and liver mass after starting the respective diets. The body weight of mice fed CDAHFD was lower compared to the control group. After 12 weeks on the diet, the body weights of the CDAHFD and control groups were 83.6 ± 7.2% and 107.5 ± 16.1% of the initial body weight, respectively (Figure 1A,C). The liver weight and ratio of liver weight to body weight at 8 and 12 weeks were significantly lower in the CDAHFD group than in the control group (Figure 1B). However, the body and liver weights of C.B17 SCID mice fed CDAHFD increased (Supplementary Figure S1A–C). Next, we examined the biochemical blood markers of hepatic function. The human albumin (h-Alb) blood level secreted by the transplanted h-heps significantly decreased in the CDAHFD group and was approximately 70% of its initial level at 12 weeks after CDAHFD feeding. The h-Alb level in the CDAHFD group was lower than the control group throughout the entire experiment (Figure 1D). Next, we assessed changes in plasma alanine aminotransferase (ALT) activity after starting the diet. Total plasma ALT activity in the CDAHFD group was transiently increased at 2 and 4 weeks, and then gradually attenuated from 6 to 12 weeks. In the control group, total ALT activity gradually increased from 8 to 12 weeks (Figure 1E). Since ALT activity was derived from h-heps and host m-heps in chimeric mice, we measured the level of human ALT1 (h-ALT1). The total ALT activity changes were consistent with the change in human ALT1 concentration (Figure 1F). Based on the results, h-heps were injured by CDAHFD feeding. In C.B17 SCID mice, total plasma ALT activity in the CDAHFD group increased at 2 weeks and was higher than in chimeric mice; the high level was maintained throughout the experiment (Supplementary Figure S1D). The replacement index was determined by immunohistochemistry using a STEM 121 antibody, a marker of human cells, in the CDAHFD and control groups at 12 weeks, and was 94.1 ± 1.1% and 98.7 ± 1.1%, respectively (Figure 1G).

### 3.2. CDAHFD Feeding Increased H-Hep Ballooning Containing Mallory–Denk Bodies and Inflammatory Cells

**Figure 2:**
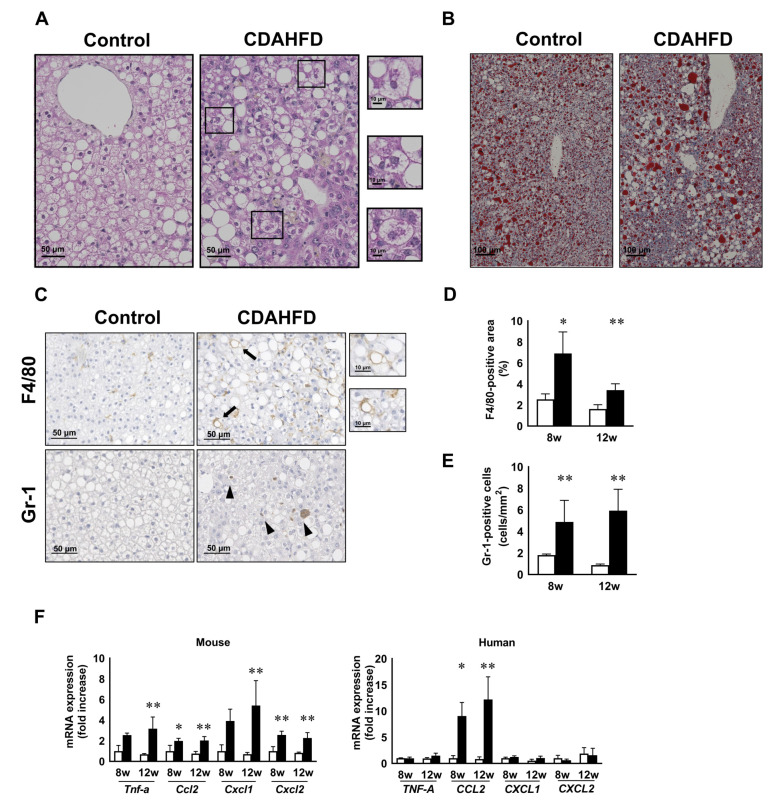
Degeneration of h-heps and inflammation in human chimeric mice after CDAHFD feeding. Representative images of H&E staining (**A**) in the liver of mice fed a control diet or CDAHFD at week 12. Scale bar: 50 μm. Areas in the squares in the CDAHFD group were magnified. Ballooning hepatocytes and Mallory–Denk-body-like aggregates were observed. Scale bar: 10 μm. Representative images of Oil Red O staining (**B**) in the liver of mice fed a control diet or CDAHFD at week 12. Scale bar: 100 mm. Representative images of immunostaining with F4/80 or Gr-1 antibody in the liver at week 12 of mice fed a control diet or CDAHFD (**C**). Scale bar: 50 μm. Areas in the squares of F4/80 in the CDAHFD group were magnified. Scale bar: 10 μm. Gr-1-positive cells were shown by arrows. The ratio of the F4/80-positive area (**D**) and number of Gr-1-positive cells in the liver of the control and CDAHFD groups at 8 or 12 weeks (**E**). Values for the F4/80-positive area and Gr-1-positive cells are represented as the mean ± standard deviation (*n* = 4, respectively). Changes in the levels of inflammation-related genes of the control and CDAHFD groups at 8 and 12 weeks (**F**). Results are expressed as the mean ratio of each value to the control group at 8 weeks. Results are represented as the mean ± standard deviation (*n* = 4 per group). Statistical comparison among multiple groups was evaluated using ANOVA, followed by Tukey’s post hoc test. * Significant difference from the control group (*p* < 0.05). ** Significant difference from the control group (*p* < 0.01).

To understand the histopathologic characteristics, we performed hematoxylin and eosin (H&E) and Oil Red O staining. Lipid accumulation was observed in the livers of the control group due to h-GH deficiency. Large-sized lipid droplets were observed in the livers of the CDAHFD group (Figure 2A,B). Significantly, ballooning hepatocytes containing Mallory–Denk body (MDB)-like aggregates appeared in the CDAHFD group at 12 weeks (Figure 2A, Table 1). However, there were no differences in the NAFLD activity score between chimeric mouse livers fed either a control diet or CDAHFD for 8 and 12 weeks (Table 1). In C.B17 SCID mice, large vacuolations and infiltrated cells were frequently observed in the CDAHFD group (Supplementary Figure S2). Next, we performed immunostaining to assess liver inflammation. The number of F4/80-positive macrophages and Gr-1-positive neutrophils increased in the CDAHFD group at 8 and 12 weeks (Figure 2C–E). Macrophages were located surrounding hepatocytes containing large lipid droplets, and crown-like structures were also observed (Figure 2C). In addition, we analyzed the expression of inflammation-related genes. Human genes were derived from h-heps, while mouse genes were derived from non-parenchymal cells or remaining m-heps, respectively. The expression of *m-Cxcl2, m-Ccl2,* and *h-CCL2* was higher in the CDAHFD-fed mice than those fed a standard diet at 8 and 12 weeks. The expression of *m-Tnfa* and *m-Cxcl1* increased at 12 weeks in the CDAHFD group compared to those fed a standard diet. There was no change in the expression of *h-TNFA, h-CXCL1,* and *h-CXCL2*, regardless of diet type (Figure 2F).

### 3.3. CDAHFD Feeding Caused Apoptosis, Proliferation of H-Heps, and Oxidative Stress

**Figure 3:**
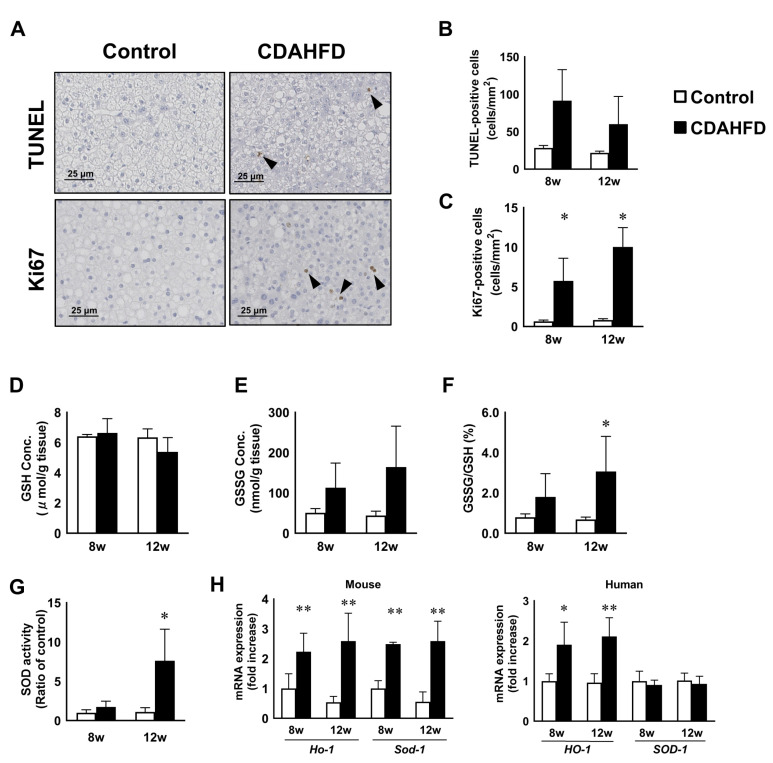
Apoptosis and proliferation of h-heps and oxidative stress in the liver of human chimeric mice after CDAHFD feeding. Representative images of TUNEL staining and immunostaining with a Ki67 antibody in the livers of the control and CDAHFD groups at 12 weeks (**A**). Positive cells are shown by arrows. Scale bar: 25 μm. The number of TUNEL- (**B**) and Ki67- (**C**) positive cells in the livers of the control and CDAHFD groups at 8 and 12 weeks. Changes in the levels of GSH, GSSG, and the ratio of GSSG to GSH of control and CDAHFD-fed mice at week 8 or 12 (**D**–**F**). Changes in the SOD activities of control and CDAHFD-fed mice at week 8 or 12 (**G**). Changes in the levels of oxidative stress-related genes of the control and CDAHFD groups at 8 and 12 weeks (**H**). Results are expressed as the mean ratio of each value to the control group at 8 weeks. Opened and closed bars show the control and CDAHFD-fed mice, respectively. Results are represented as the mean ± standard deviation (*n* = 4 per group). Statistical comparison among multiple groups was evaluated using ANOVA, followed by Tukey’s post hoc test. * Significant difference from the control group (*p* < 0.05). ** Significant difference from the control group (*p* < 0.01).

Apoptosis of hepatocytes is observed in simple steatosis and throughout the progression to NASH [35,36]. We assessed apoptotic cell death in the livers of chimeric mice after CDAHFD feeding with a terminal deoxynucleotidyl transferase dUTP nick end labeling (TUNEL) assay. At 8 and 12 weeks, the prevalence of TUNEL-positive apoptotic cells in the CDAHFD group were higher compared to the control group although the difference was not statistically significant (Figure 3A,B). However, the liver is a highly regenerative organ with resilience against damage. We performed immunostaining using a Ki67 antibody, a human cell-specific proliferation marker, to confirm cell proliferation in the CDAHFD group. The number of Ki67-positive cells also increased 10- and 15-fold at 8 and 12 weeks in the CDAHFD group, respectively (Figure 3A,C). Next, we examined the glutathione levels and SOD activity, as indicators of oxidative stress, in the livers of chimeric mice after CDAHFD feeding. There was no change in the concentration of GSH, regardless of diet type (Figure 3D). The level of GSSG tended to increase at 8 and 12 weeks in the CDAHFD group (Figure 3E). Accordingly, the ratio of GSSG to GSH, was higher in CDAHFD group at 12 weeks significantly (Figure 3F). The SOD activity was enhanced at 12 weeks in the CDAHFD group (Figure 3G). Furthermore, the expression of m-Ho-1, m-Sod-1 and h-HO-1 were higher in the CDAHFD-fed mice compared to those fed a standard diet at 8 and 12 weeks. There was no change in the expression of h-SOD-1 (Figure 3H).

### 3.4. CDAHFD Feeding Caused Liver Fibrosis Accompanied by Increases of Fibrotic Gene Expression and Mac-2 Binding Protein Concentration in Mouse Sera

**Figure 4:**
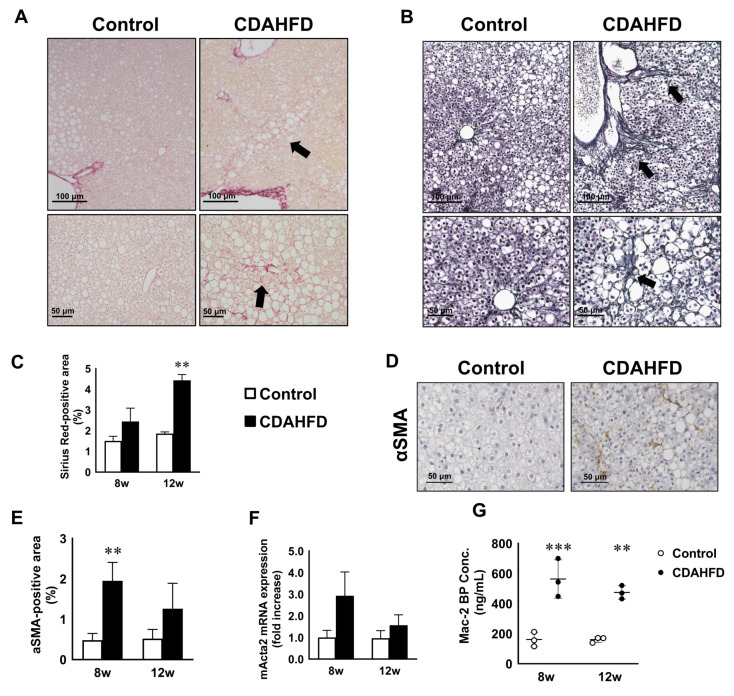

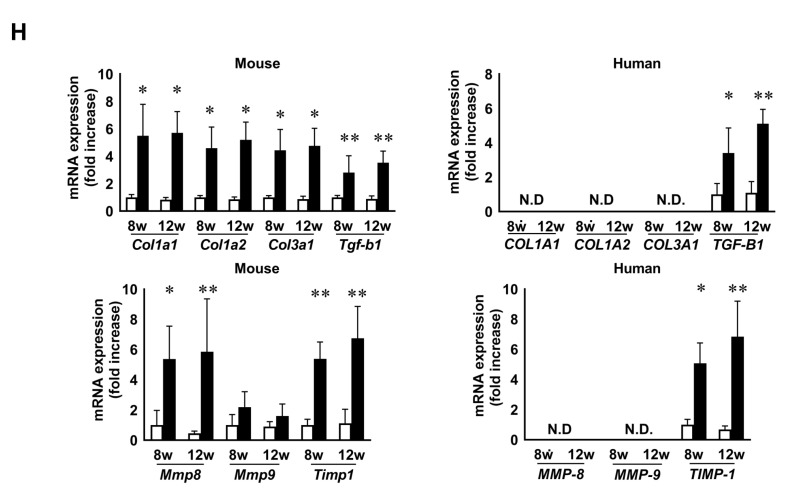
Fibrosis in human chimeric mice after CDAHFD feeding. Representative images of Sirius Red (**A**) and silver (**B**) staining in the livers of the control and CDAHFD groups at 12 weeks. Scale bar: 100 μm (upper), 50 μm (lower). The ratio of the Sirius red-positive area in the livers of the control and CDAHFD groups at 8 or 12 weeks (**C**). Immunostaining with αSMA antibody in the livers of the control and CDAHFD groups at 12 weeks (**D**). Scale bar: 50 μm. The ratio of the αSMA positive area in the livers of the control and CDAHFD groups at 8 or 12 weeks (**E**). Results are represented as the mean ± standard deviation (*n* = 3). Changes in the levels of mouse *Acta2* (**F**) in the livers of the control and CDAHFD groups at 8 and 12 weeks. Results are expressed as the mean ratio of each value to the control group at 8 weeks. Plasma Mac-2 binding protein levels in the sera of control and CDAHFD-fed mice at 8 and 12 weeks (**G**). Changes in the levels of fibrosis-related gene expression (**H**) in the livers of the control and CDAHFD groups at 8 and 12 weeks. Results are expressed as the mean ratio of each value to the control group at 8 weeks. Statistical comparison among multiple groups was evaluated using ANOVA, followed by Tukey’s post hoc test. * Significant difference from the control group (*p* < 0.05). ** Significant difference from the control group *(p* < 0.01). *** Significant difference from the control group (*p* < 0.001).

Hepatic fibrosis was caused by the infiltration of inflammatory cells to regenerate damaged hepatocytes. Hepatic fibrosis after CDAHFD feeding was evaluated by Sirius Red and silver stains. Perisinusoidal and pericellular Sirius Red-positive collagen fibers were observed at 8 and 12 weeks in the CDAHFD-fed group. Moreover, these fibers were formed surrounding degenerating hepatocytes. However, in the livers of control mice, Sirius Red-positive fibers were limited only to the portal area or central vein. Positive staining with Sirius Red was significantly higher in the CDAHFD group at 12 weeks compared to the control group (Figure 4A,C). In terms of the control group, reticular fibers were not observed in the hepatic parenchyma except for around the blood vessels. In the liver of the CDAHFD group, reticular fibers were observed throughout the hepatic parenchyma, extending towards the central or portal veins (Figure 4B). Furthermore, the fibers were milder in chimeric mice compared to CDAHFD-fed C.B17 SCID mice (Supplementary Figure S3). Next, we determined the activation of hepatic stellate cells (HSC) which play a pivotal role in liver fibrosis. α–smooth muscle actin (αSMA)-positive activated HSCs were higher in the CDAHFD group, with a corresponding significant increase in αSMA-positive liver area at 8 weeks compared to the control group. The increase persisted until week 12, although there was no statistical significance (Figure 4D,E). The expression of *m-Acta2* tended to increase after CDAHFD feeding (Figure 4F). Recently, plasma Mac-2 binding protein glycosylation isomer (M2BPGi) was reported as a novel marker of liver fibrosis [37]. Therefore, we investigated the change in the plasma Mac-2BP concentration in both groups. Plasma Mac-2BP levels were higher in the CDAHFD group at 8 and 12 weeks, compared to the control group (Figure 4G). We further analyzed fibrosis-related genes. The expression levels of *m-Col1a1, m-Col1a2,* and *m-Col3a1* in the CDAHFD group were higher compared to the control group at weeks 8 and 12. In addition, the expression of pro-fibrotic cytokines *h-TGF-B1* and *m-Tgf-b1* were higher at 8 and 12 weeks compared to the control group. The expression of *m*-*Mmp-8*, a fibrolytic gene, was upregulated by CDAHFD feeding, but *m*-*Timp-1* and *h*-*TIMP-1*, endogenous inhibitor of MMPs, was also upregulated. Human collagen and MMP genes were not detected (Figure 4H).

### 3.5. Reversion to a Control Diet Improved Steatosis and H-Hep Ballooning, But Not Fibrosis

We examined the changes in NASH pathology after returning to a standard diet after the development of NASH (Supplementary Figure S5A). When reverting to the standard diet from CDAHFD, body weight increased. However, there was no change in liver weight (Supplementary Figure S5B–D). Blood h-Alb and serum ALT activities were restored after returning to a standard diet (Supplementary Figure S5E,F). In addition, histological analysis showed that standard diet improved steatosis and ballooning degeneration, but not fibrosis (Supplementary Figure S5G–I).

### 3.6. Prophylactic Treatment of Elafibranor Prevented Steatosis, H-Hep Ballooning, and Fibrosis While the Therapeutic Treatment Improved Steatosis and H-Hep Ballooning, But Not Fibrosis

**Figure 5:**
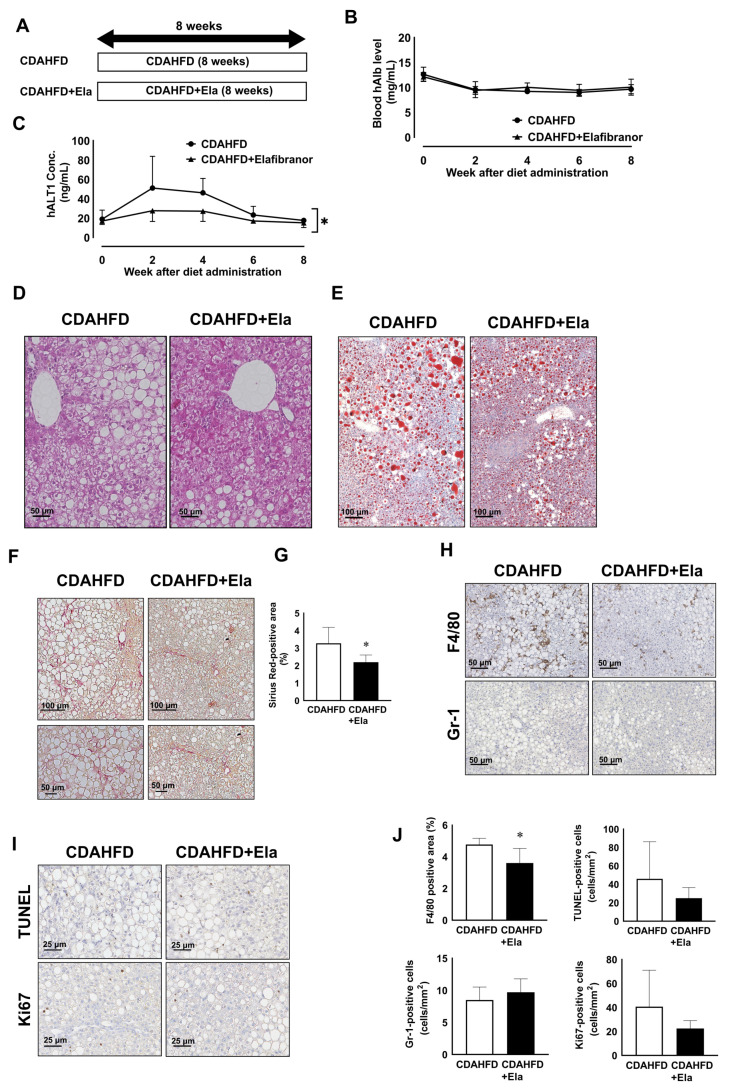
Elafibranor ameliorated steatosis, ballooning and fibrosis. Schematic figure for the prevention study with Elafibranor using CDAHFD-fed chimeric mice (**A**). Time course changes in the blood h-Albumin (**B**) and human specific ALT1 levels (**C**) of the CDAHFD with (closed triangle) or without (closed circle) Elafibranor groups. Representative images of H&E staining in the livers of CDAHFD-fed mice with or without Elafibranor (**D**). Scale bar: 50 μm. Representative images of Oil Red O staining (**E**) in the liver of mice fed a control diet or CDAHFD at week 12. Scale bar: 100 μm. Representative images of Sirius Red staining in the livers of CDAHFD-fed mice with or without Elafibranor (**F**). Scale bar: 100 μm (upper), 50 μm (lower). The ratio of Sirius Red-positive area in the livers of CDAHFD-fed mice with or without Elafibranor (**G**). Results are represented as the mean ± standard deviation (*n* = 4). Representative images of F4/80 and Gr-1 immunostaining in the livers of CDAHFD-fed mice with or without Elafibranor (**H**). Scale bar: 50 μm. Representative images of Ki67 antibody and TUNEL staining in the livers of CDAHFD-fed mice with or without Elafibranor (**I**). Scale bar: 25 μm. The ratio of the F4/80-positive area and number of Gr-1-, TUNEL-, and Ki67-positive cells in the livers of CDAHFD-fed mice with or without Elafibranor (**J**). Results are expressed as the mean ± standard deviation (*n* = 4). Differences between the two groups were evaluated statistically using an unpaired Student’s *t* test. * Significant difference from the CDAHFD group (*p* < 0.05).

Furthermore, we conducted a drug efficacy study for NASH using Elafibranor to determine whether this model could be used for the processes of drug development. We investigated the prevention effects of Elafibranor on NASH progression (Figure 5A). The blood h-Alb level was not changed with Elafibranor treatment (Figure 5B). However, the peak serum hALT1 concentration in the Elafibranor group was lower than that of the untreated CDAHFD group (Figure 5C). In addition, H&E and Oil Red O staining revealed that the number of hepatic macro lipid droplets was reduced with Elafibranor treatment (Figure 5D,E). Furthermore, Sirius Red-positive fibers were decreased by Elafibranor treatment (Figure 5F,G). Elafibranor prevented an increase in the F4/80-positive area, but the number of Gr-1-positive neutrophils was not changed (Figure 5H,J). TUNEL-positive apoptotic cells and Ki67-positive proliferating cells tended to decrease with Elafibranor treatment (Figure 5I,J).

**Figure 6:**
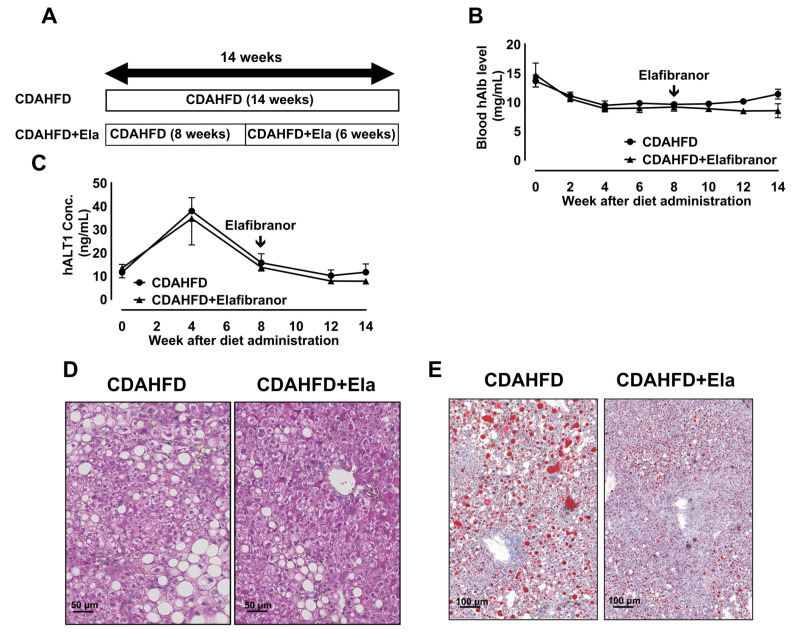

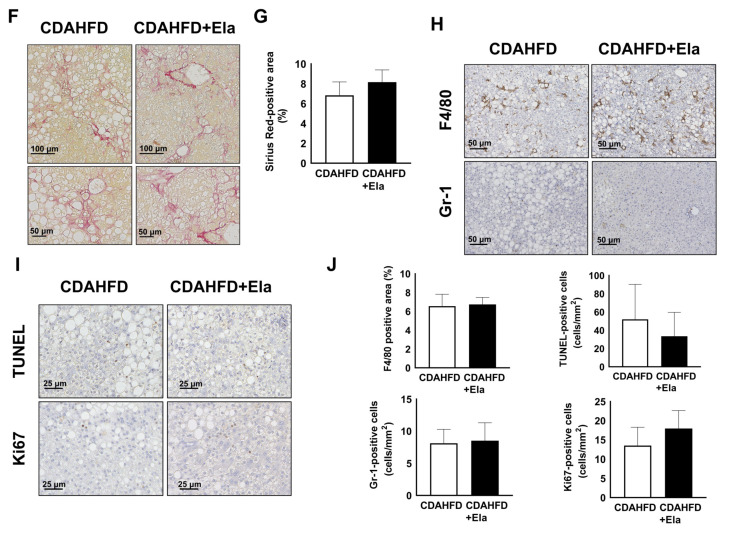
Elafibranor prevented the progression of steatosis and ballooning but not fibrosis. Schematic figure in the therapeutic study with Elafibranor using CDHFD-fed chimeric mice (**A**). Time course changes in the blood h-Albumin (**B**) and human specific ALT1 levels (**C**) of the CDAHFD with (closed triangle) or without (closed circle) Elafibranor. Representative images of H&E staining in the livers of CDAHFD-fed mice with or without Elafibranor (**D**). Scale bar: 50 μm. Representative images of Oil Red O staining (**E**) in the liver of CDAHFD with or without Elafibranor. Scale bar: 100 μm. Representative images of Sirius Red staining in the liver of CDAHFD with or without Elafibranor (**F**). Scale bar: 100 μm (upper), 50 μm (lower). The ratio of the Sirius Red-positive area in the livers of CDAHFD-fed mice with or without Elafibranor (**G**). Results are represented as the mean ± standard deviation (*n* = 4). Representative images of F4/80 and Gr-1 immunostaining in the livers of CDAHFD-fed mice with or without Elafibranor (**H**). Scale bar: 50 μm. Representative images of Ki67 antibody and TUNEL staining in the livers of CDAHFD-fed mice with or without Elafibranor (**I**). Scale bar: 25 μm. The ratio of the F4/80-positive area and number of Gr-1-, TUNEL-, and Ki67-positive cells in the livers of CDAHFD-fed mice with or without Elafibranor (**J**). Results are expressed as the mean ± standard deviation (*n* = 4). Differences between the two groups were evaluated statistically using an unpaired Student’s t test.

Next, we examined the therapeutic effects on developed NASH (Figure 6A). Blood h-Alb and serum hALT1 levels were not changed after Elafibranor treatment (Figure 6B,C). Elafibranor markedly reduced hepatic steatosis and ballooning degeneration of h-heps (Figure 6D,E). Accordingly, the NAFLD activity score was reduced by Elafibranor treatment (Table 2). However, hepatic fibrosis, inflammation, and hepatocytic apoptosis and proliferation were not affected by Elafibranor treatment (Figure 6F–J).

## 4. Discussion

Many studies have tried to understand the pathology of NAFLD/NASH through animal models in order to develop therapeutics for the disease [38,39]. Although drug candidates have been found in rodent models, to date, there are still no approved drugs. One of the reasons is the lack of an appropriate animal model of NASH that resolves the species differences between humans and rodents. Gene expression levels of hepatocytes from chimeric mice were compared with those of hepatocytes from human livers by microarray analysis. We observed that 82% of transcripts were expressed in both hepatocytes within a two-fold range difference [40]. Using LC-MS/MS, protein expression levels of CYP, UGT, and transporters were also compared between ten human livers and six PXB-mouse livers transplanted with two donors. The protein expression levels were all within a 4-fold range difference [41]. Chimeric mice with human livers have human gene and protein expressions including human-type drug metabolizing enzymes [15,16], lipoproteins [42] and nuclear receptors targeted against drugs [26,43], and are thus useful for elucidating the mechanism of NAFLD/NASH and developing new drugs. In this study, we developed a NASH mouse model using chimeric mice with highly repopulated h-heps (>90%) to reduce the effects of mouse hepatocytes.

First, we evaluated the physiologic changes of human liver chimeric mice after CDAHFD feeding. The body and liver weights of the CDAHFD group decreased compared with the control group. Conversely, the body and liver weights of C.B17 SCID mice fed CDAHFD increased by the accumulation of fat (Supplementary Figures S1B,C and S2). Our previous study demonstrated that chimeric mice with h-heps spontaneously developed hepatic steatosis after transplantation due to h-GH deficiency because the h-GH receptors (h-GHR) on the h-heps of chimeric mice do not react with rodent GH [44]. GH signaling, which plays an important role in lipid metabolism regulation [18], does not function properly. Therefore, the livers of chimeric mice with notable hepatic enlargement may have been in a NAFLD state before feeding, which may be an aggravating factor in this model. Furthermore, we have shown that human hepatocytes are slower to proliferate than those of rodents [45]. There is a possibility that the capacity of regeneration against liver damage in chimeric mice with h-heps may be slow. Thus, the decrease in the liver weight of human chimeric mice after CDAHFD feeding may be caused by an imbalance between cell death and regeneration.

Next, we conducted biochemical analysis of hepatic function after CDAHFD feeding. In human patients, the serum Alb level significantly decreases when the severity of NAFLD increases [46]. The h-Alb level in human liver chimeric mice decreased with CDAHFD feeding compared to the control group. The decrease in h-Alb level (70%) was more severe than a decrease in the replacement index of h-heps (95%). These results suggested that albumin synthesis in human hepatocytes decreased due to CDAHFD feeding.

To further understand the pathology of the NASH model using human liver chimeric mice, we performed histological analysis after CDAHFD feeding. In the livers of human chimeric mice fed CDAHFD, hepatocyte degeneration, such as ballooning and MDB-like aggregates, were observed in the h-heps but not m-heps areas. Furthermore, these pathological features could not be clearly observed in the liver of C.B17 SCID mice fed CDAHFD. Ballooning hepatocytes in the livers of humanized chimeric mouse fed CDAHFD were enlarged, rounded, and swollen-appearing, similar to that observed in human NASH patients [47,48]. Therefore, it suggests that the ballooning hepatocytes observed in this model can be useful as an indicator of human NASH pathology.

Next, we examined the presence of inflammation after CDAHFD feeding. We confirmed the increased inflammatory cells and responses by immunostaining and qRT-PCR. However, the infiltration of inflammatory cells was not significant in the NAS score determined by H&E staining probably due to the use of SCID background mice. Additionally, peri-sinusoidal and pericellular fibrosis extending from the portal and/or central veins were observed in the liver of human-liver chimeric mice fed CDAHFD. HSC activation was also observed in chimeric mice after CDAHFD feeding (Supplementary Figure S4). Transforming growth factor-β1 (TGF-β1) plays an important role in HSC activation [49] and is highly homologous between humans and mice. Our results showed that TGF-β1 expression increased not only in m-heps or m-non-parenchymal cells, but also in h-heps. These results suggest that TGF-β secreted by h-heps partially contributed to the activation of mouse HSCs. The establishment of this crosstalk indicates a flow from h-heps injury to mouse HSC activation and the subsequent development of fibrosis. In contrast, chimeric mice with human livers do not have compatible cellular communication between h-heps to non-parenchymal cells and/or endocrine factors from mouse organs. For example, it is known that mice with humanized livers have abnormal bile acid signaling due to homology differences of fibroblast growth factor (FGF) 19, the human ortholog of mouse FGF15. Because m-FGF15 cannot react with h-FGFR4, the downstream genes of *h-FGFR4*, *CYP7A1* is upregulated, resulting in an increase in bile acid synthesis in h-heps [50]. As mentioned above, m-GH released from the mouse pituitary gland does not react with h-GHR in implanted h-heps [18]. Incompatibilities other than m-FGF15/h-FGFR4 and m-GH/h-GHR may have resulted in the mild responses of inflammation or fibrosis in chimeric mice fed a CDAHFD compared to C.B17 SCID mice fed a CDAHFD.

Weight loss due to lifestyle changes (diet and exercise) has been considered the only effective treatment for NAFLD/NASH. Many reviews showed that lifestyle interventions reduced ALT activity and the NAFLD activity score consisting of steatosis, ballooning, and inflammation [51], but not hepatic fibrosis [52]. We also examined the changes in NASH pathology when returning to a normal diet after the development of NASH. Consistent with human studies, our results also showed that returning to a standard diet for only 4 weeks significantly improved steatosis and ballooning but not fibrosis. These results suggest that only lifestyle improvements are insufficient for the treatment of hepatic fibrosis in NASH.

Finally, we conducted a pharmacological study to determine whether this model is useful for developing new drugs against NASH. Drug candidates targeting nuclear receptors, including PPARs, have been recently developed. There are three subtypes of PPAR: PPARα, PPARδ, and PPARγ. Among them, PPARα is expressed at high levels in hepatocytes and is thus considered to play a central role in the metabolism and effects of xenobiotics, including drugs [22]. There is also the issue of interspecies differences in the activity of this PPAR subtype. Their ligand-binding domains exhibit a lower degree of sequence identity (~65%); PPARα agonists induce seemingly different responses in rodents and humans [53]. Previously, to characterize the species-specific action of PPARs, we evaluated the in vivo effect of fenofibrates on peroxisome proliferation in chimeric mice with human hepatocytes [26,27]. The agonist fenofibrate was examined for changes at the histological level and related gene and protein expression. As a result, we defined the generally recognized species-specific effects of the PPARα agonist and thus supported the notion that rodent data on PPARα-induced hepatocarcinogenesis cannot be accurately extrapolated to human data. Another example of rodent and human species difference of nuclear receptors is that of the constitutive androstane receptor (CAR) activator, sodium phenobarbital (NaPB). NaPB has mitogenic effects in rat and mouse hepatocytes in both in vitro and in vivo studies. However, this compound does not stimulate growth in cultured human hepatocytes and in in vivo studies performed in chimeric mice with human hepatocytes, suggesting that NaPB-induced rodent liver tumor formation is not relevant for humans [43]. From these results, we believe that the NASH model using humanized livers is an ideal model to elucidate the efficacy and toxicity of drug candidates targeting human genes or proteins.

Elafibranor is a dual PPAR α/δ agonist. As shown above, PPARα agonists induce different responses in rodents and humans. PPARδ is expressed in Kupper cells, stellate cells, and hepatocytes, and KD3010, a PPARδ agonist, has been shown to exhibit hepatoprotective and antifibrotic effects in a carbon-tetrachloride (CCl4)-induced fibrotic mouse model [54]. In clinical trials, Elafibranor significantly improved steatosis, ballooning, and inflammation in NASH patients. Furthermore, some patients were observed to have a reduction of fibrosis [30]. Therefore, Elafibranor was chosen because its efficacy can be compared in a NASH model using human chimeric mice. Our results showed that Elafibranor treatment improved steatosis. Furthermore, preventive treatment with Elafibranor mitigated the increase in serum hALT1 levels and partially suppressed inflammatory responses and fibrosis progression. However, therapeutic treatment with Elafibranor did not affect symptoms other than steatosis and ballooning. Liver damage and HSC activation of human liver chimeric mice peaked at 2 to 4 weeks after CDAHFD feeding (Supplementary Figure S5). Hence, treatment with Elafibranor 8 weeks after CDAHFD feeding may not suppress liver damage. However, a preventative treatment with Elafibranor may suppress liver injury at 2 to 4 weeks after CDAHFD feeding by reducing subsequent inflammation, HSC activation, and fibrosis. The effect of Elafibranor on human chimeric mice fed a CDAHFD was consistent with clinical trial results. It has been shown that this model is useful for pharmacological studies in the development of NASH therapies. Ballooning hepatocytes may be useful as a marker for drug efficacy because their morphology is similar to that of human NASH. However, it may not be suitable for evaluating anti-inflammatory effects, as human liver chimeric mice are derived from the SCID background.

## 5. Conclusions

Our CDAHFD-induced NASH model using human liver chimeric mice is a novel humanized mouse model of NASH, with the pathophysiological features commonly observed in mild and/or the early stages of human NASH. This model may be useful in exploring new drugs targeting human genes or proteins and biomarkers in the early stages of human NASH.

## Figures and Tables

**Table 1:** NAFLD activity score in chimeric mouse livers fed a control diet and CDAHFD for 8 and 12 weeks.

		Animal No.	Steatosis (%)	Ballooning	Lobular Inflammation	NAFLD Activity Score
control	8 w	1	60	N.D.	±	3
		2	50	N.D.	N.D.	2
		3	40	N.D.	+	3
	12 w	4	50	N.D.	±	2
		5	50	N.D.	N.D.	2
		6	50	N.D.	N.D.	2
		7	50	N.D.	N.D.	2
CDAHFD	8 w	8	50	N.D.	N.D.	2
		9	40	N.D.	±	3
		10	40	N.D.	N.D.	2
		11	40	N.D.	N.D.	2
	12 w	12	50	+	±	3
		13	50	+	N.D.	2
		14	40	+	N.D.	2

N.D.: not detected

**Table 2:** NAFLD activity score in chimeric mouse livers fed a CDAHFD (14 w) with or without Elafibranor.

	Animal No.	Steatosis (%)	Ballooning	Lobular Inflammation	NAFLD Activity Score
CDAHFD	A	60	±	±	4
	B	60	±	N.D.	3
	C	60	+	N.D.	3
	D	50	+	N.D.	3
CDAHFD + Elafibranor	E	30	+	N.D.	2
	F	30	N.D.	N.D.	1
	G	15	N.D.	N.D.	1
	H	20	N.D.	±	2
	I	20	N.D.	±	2
	J	33	N.D.	±	2

N.D: Not detected.

## Data Availability

The data presented in this study are all contained within the main body of this article.

## Supplementary Materials

The following are available online at https://www.mdpi.com/article/10.3390/biomedicines9111647/s1, Supplementary Figure S1: Changes in the physical and biochemical profiles of C.B17 SCID and chimeric mice with h-heps after CDAHFD feeding. Supplementary Figure S2: Histological changes in C.B17 SCID mice at 14 weeks after CDAHFD feeding. Supplementary Figure S3: Comparison of changes in inflammation and fibrosis-related gene expression in the C.B17 SCID and chimeric mice with h-heps at 14 weeks after CDAHFD feeding. Supplementary Figure S4: Activation of hepatic stellate cells in human chimeric mice after CDAHFD feeding. Supplementary Figure S5: Changes in NASH pathology when retuning to a standard diet after developed NASH. Supplementary Table S1: Antibodies used for immunohistochemistry in this study. Supplementary Table S2: Primers used for qRT-PCR in this study.
