# Supplementary material for: Estimating Drug Efficacy with a Diet-Induced NASH Model in Chimeric Mice with Humanized Livers
Source: Biomedicines. 2021 Nov 9;9(11):1647. doi: 10.3390/biomedicines9111647 (PMC8615377; doi:10.3390/biomedicines9111647)
Supplement: Supplementary file 1 [file biomedicines-09-01647-s001.zip › Supplementary Figure S1.pdf]

Supplementary Figure S1

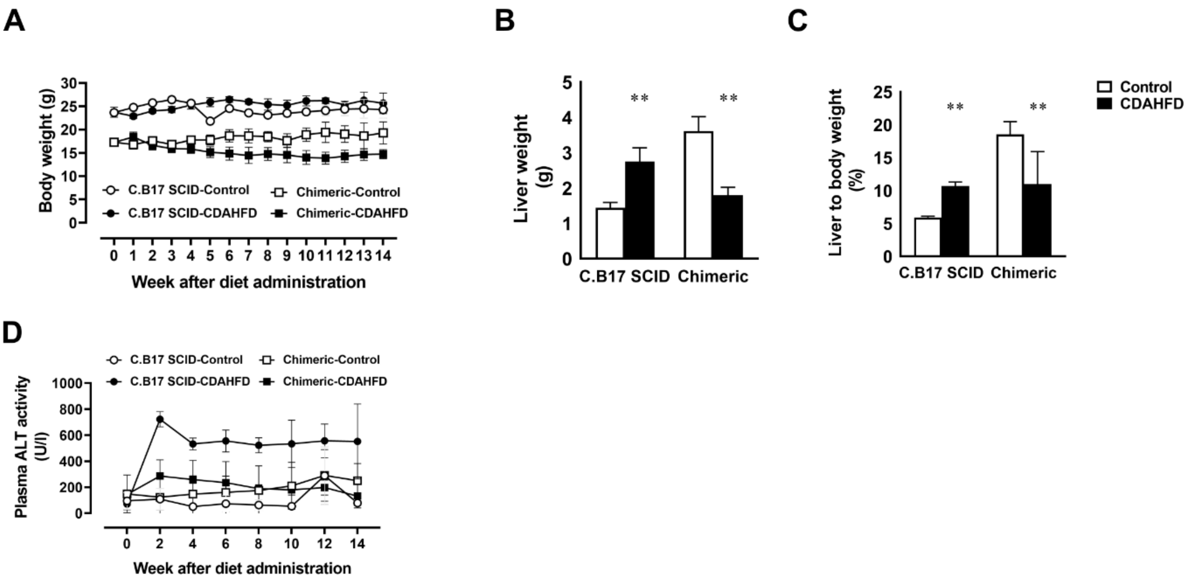

Supplementary Figure S1. Changes in the physical and biochemical profiles of C.B17 SCID and chimeric mice with h-heps after CDAHFD feeding. Time course changes in the body weights of the C.B17 SCID mice (circle) or chimeric mice (square) in the control (open circle and square) and CDAHFD groups (closed circle and square) (A). The liver weight (B) and the ratio of liver weight to body weight (C) of the mice in the control and CDAHFD groups at 14 weeks. The open and closed bars show the control and CDAHFD-fed mice, respectively. The time course changes in the level of plasma ALT activity (D) of the control and CDAHFD groups. Results are represented as the mean ±standard deviation (n = 4 per group). **Significant difference from the control group (*p* <0.01).
