# Supplementary material for: Estimating Drug Efficacy with a Diet-Induced NASH Model in Chimeric Mice with Humanized Livers
Source: Biomedicines. 2021 Nov 9;9(11):1647. doi: 10.3390/biomedicines9111647 (PMC8615377; doi:10.3390/biomedicines9111647)
Supplement: Supplementary file 1 [file biomedicines-09-01647-s001.zip › Supplementary Figure S2.pdf]

Supplementary Figure S2

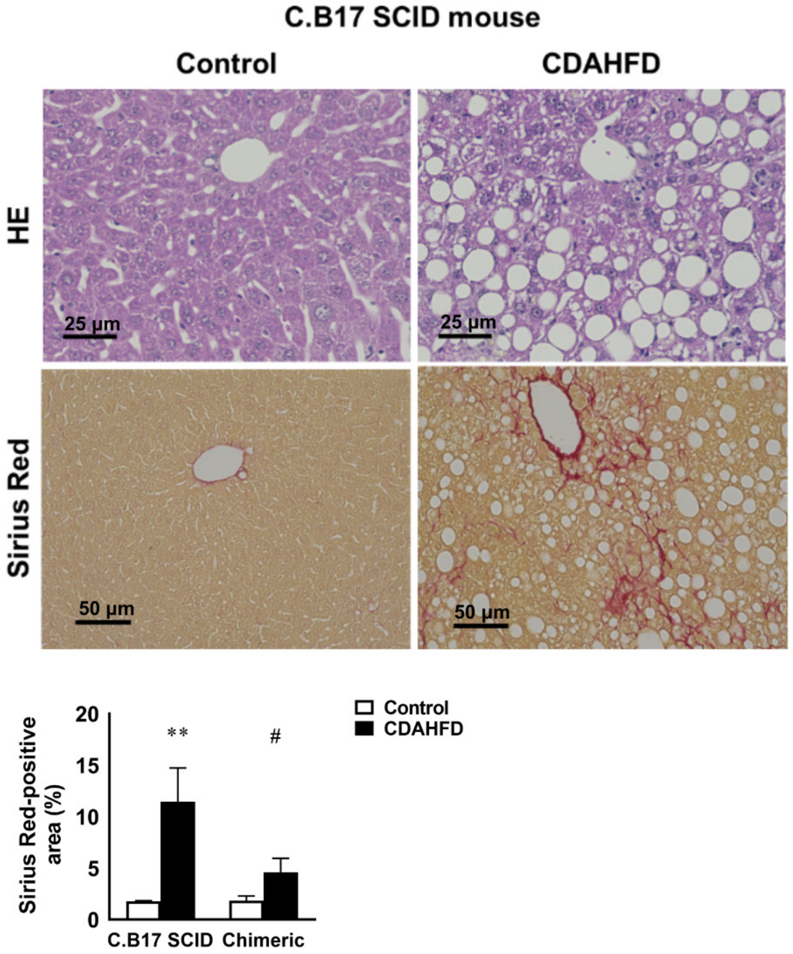

Supplementary Figure S2 Histological changes in C.B17 SCID mice at 14 weeks after CDAHFD feeding. Representative images of H&E staining (upper panel) and Sirius Red staining (lower panel) in the livers of standard diet or CDAHFD fed C.B17 SCID mouse (n = 4 per group). Scale bar: 25 µm (upper panel) or 50 µm (lower panel). The ratio of the Sirius Red-positive area in the livers of control or CDAHFD fed mice at 14 weeks. The open and closed bars show the control and CDAHFD-fed mice, respectively (n=4 per group). **Significant difference from the control group (p <0.01). [#]Significant difference from the C.B17 SCID mice fed CDAHFD (p <0.05).
