# Supplementary material for: Estimating Drug Efficacy with a Diet-Induced NASH Model in Chimeric Mice with Humanized Livers
Source: Biomedicines. 2021 Nov 9;9(11):1647. doi: 10.3390/biomedicines9111647 (PMC8615377; doi:10.3390/biomedicines9111647)
Supplement: Supplementary file 1 [file biomedicines-09-01647-s001.zip › Supplementary Figure S3.pdf]

Supplementary Figure S3

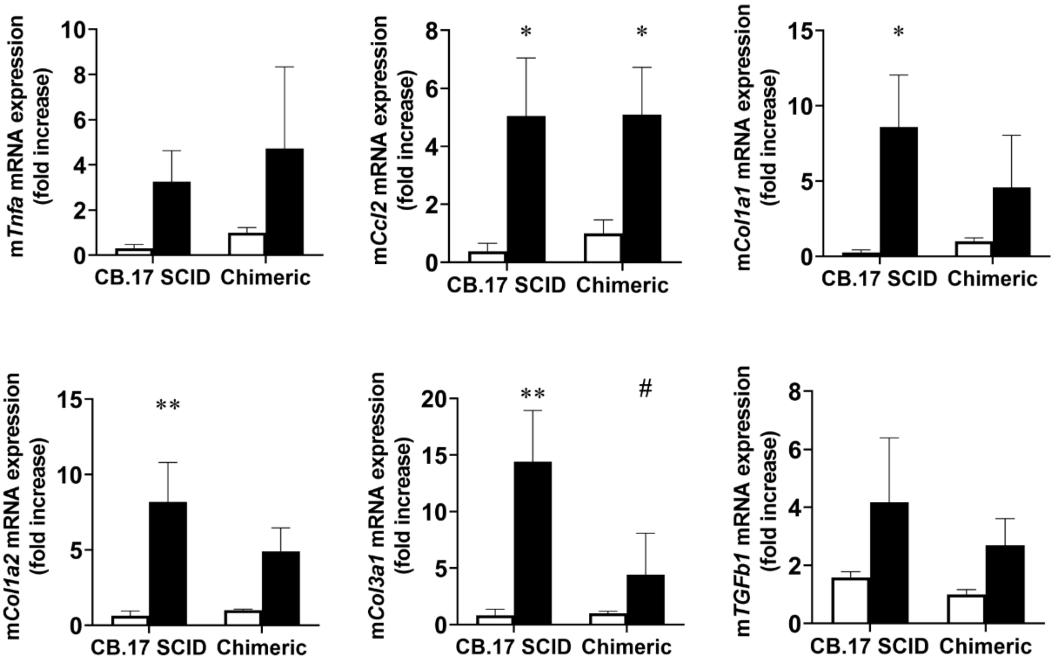

Supplementary Figure S3. Comparison of changes in inflammation and fibrosis-related gene expression in the C.B17 SCID and chimeric mice with h-heps at 14 weeks after CDAHFD feeding. Changes in the levels of inflammation and fibrosis-related gene expressions in the C.B17 SCID and chimeric mice with h-heps after CDAHFD feeding. Results are represented as the mean ratio of each value for standard diet fed chimeric mice ± standard deviation (n=4 per group). Statistical comparison among multiple groups was evaluated using an ANOVA, followed by Tukey's post hoc test. *Significant difference from the control group ($p < 0.05$). **Significant difference from the control group ($p < 0.01$). #Significant difference from the C.B 17 SCID mice fed CDAHFD ($p < 0.05$).
