# Supplementary material for: Estimating Drug Efficacy with a Diet-Induced NASH Model in Chimeric Mice with Humanized Livers
Source: Biomedicines. 2021 Nov 9;9(11):1647. doi: 10.3390/biomedicines9111647 (PMC8615377; doi:10.3390/biomedicines9111647)
Supplement: Supplementary file 1 [file biomedicines-09-01647-s001.zip › Supplementary Figure S4.pdf]

Supplementary Figure S4

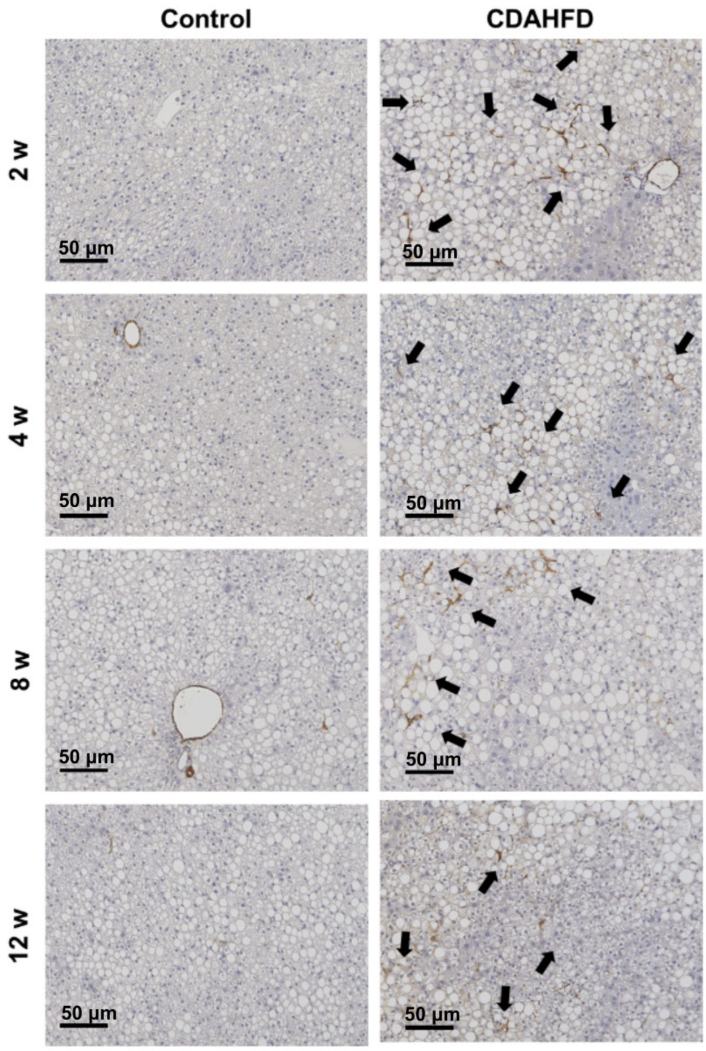

Supplementary Figure S4. Activation of hepatic stellate cells in human chimeric mice after CDAHFD feeding. Representative images of immunostaining with a αSMA antibody of the livers of the control and CDAHFD groups at 2, 4, 8, and 12 weeks (n=3 per group). Scale bar: 50 µm.
