# Supplementary material for: Estimating Drug Efficacy with a Diet-Induced NASH Model in Chimeric Mice with Humanized Livers
Source: Biomedicines. 2021 Nov 9;9(11):1647. doi: 10.3390/biomedicines9111647 (PMC8615377; doi:10.3390/biomedicines9111647)
Supplement: Supplementary file 1 [file biomedicines-09-01647-s001.zip › Supplementary Figure S5.pdf]

Supplementary Figure S5

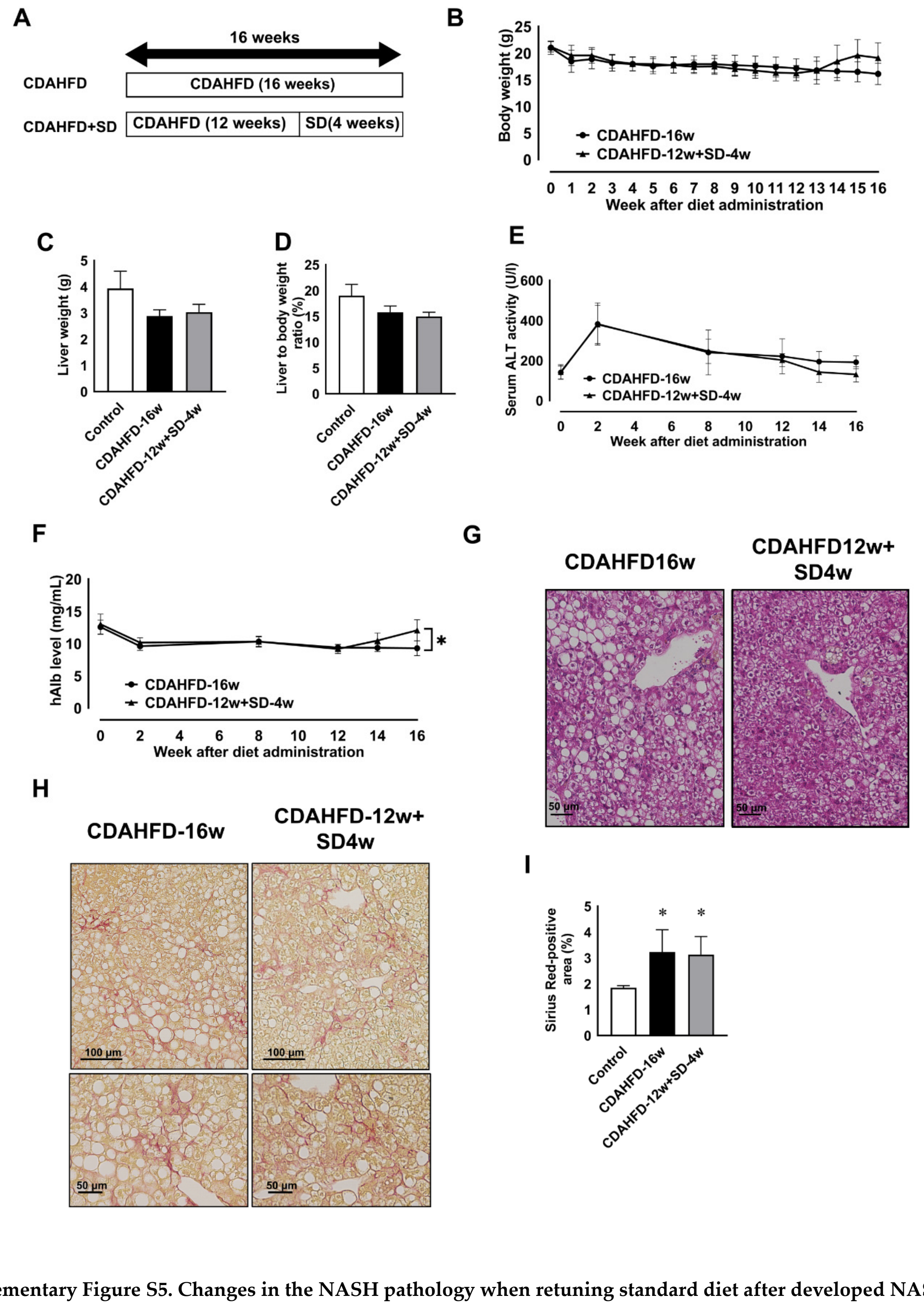

Supplementary Figure S5. Changes in the NASH pathology when retuning standard diet after developed NASH. Schematic figure for the experimental design when chimeric mice fed with CDAHFD returning to standard diet (A). Chimeric mice fed CDAHFD for 16 weeks represent as CDAHFD-16w group. Chimeric mice fed CDAHFD for 12 weeks and then switched to standard diet represent CDAHFD-12w+SD-4w group. Time course changes in the body weight of CDAHFD-16w group and CDAHFD12w+SD-4w group (B). The liver weight (C) and the ratio of liver weight to body weight (D) in control, CDAHFD and CDAHFD12w+SD-4w group. white, black, and gray bars showed control, CDAHFD-16w and CDAHFD12w+ SD-4w group, respectively. Time course changes in the level of serum ALT activity (E) and blood human Albumin level (F) of CDAHFD-16w

group and CDAHFD12w+SD-4w group. Representative images of H&E staining in the liver of mouse in CDAHFD-16w group or CDAHFD12w+SD-4w group (G). Scale bar; 50 μm. Representative images of Sirius Red staining in the liver of mouse in CDAHFD-16w group and CDAHFD12w+SD-4w group (H). Scale bar; 100 μm (upper), 50 μm (lower). The ratio of Sirius Red-positive area in the liver of mouse in each group (I). Results are expressed as the mean ± standard deviation (n = 4). Statistical comparison among multiple groups was evaluated by ANOVA, followed by Tukey's post hoc test. *Significant difference from the control group ($p < 0.05$).
