# Supplementary material for: Estimating Drug Efficacy with a Diet-Induced NASH Model in Chimeric Mice with Humanized Livers
Source: Biomedicines. 2021 Nov 9;9(11):1647. doi: 10.3390/biomedicines9111647 (PMC8615377; doi:10.3390/biomedicines9111647)
Supplement: Supplementary file 1 [file biomedicines-09-01647-s001.zip › Supplementary methods.pdf]

Estimating drug efficacy with a diet-induced NASH model in chimeric mice with humanized livers

Running head: NASH model using human liver chimeric mice

Keishi Kisoh[1$], Go Sugahara[1$], Yuko Ogawa[1], Suzue Furukawa[1], Yuji Ishida[1,2], Takeshi Okanoue[3], Michinori Kohara[4], Chise Tateno[1,2*]

[$]These authors have equally contributed to this study.

Affiliation

[1] Research and Development Department, PhoenixBio Co., Ltd., 3-4-1 Kagamiyama, Higashihiroshima, 739-0046, Japan.

[2] Research Center for Hepatology and Gastroenterology, Hiroshima University, 1-2-3 Kasumi, Minami-ku, Hiroshima 734-8551, Japan

[3]Department of Gastroenterology and Hepatology, Saiseikai Suita Hospital, 1-2 Kawazonocho, Suita, 564-0013, Japan

[4] Department of Microbiology and Cell Biology, Tokyo Metropolitan Institute of Medical Science, Setagaya-ku, Tokyo 156-8506, Japan

Address for Correspondence:

Chise Tateno

Research and Development Department, PhoenixBio Co., Ltd., 3-4-1 Kagamiyama, Higashi-hiroshima, 739-0046, Japan.

Tel: +81-82-431-0016

E-mail: chise.mukaidani@phoenixbio.co.jp

Supplementary Methods

Generation of a CDAHFD-induced NASH model using C.B17 SCID mice

Chimeric mice (15 to 18-week-old, male) with h-heps {(IVT-JFC: 1-year-old Caucasian male, purchased from Bio-IVT (Westbury, NY, USA)} and C.B17 SCID mice (6-week-old, male, Charles River Japan, Yokohama, Japan) were fed a CDAHFD or standard diet for 14 weeks.

Restoration study

Study design is shown in supplemental Figure 5A. The chimeric mice were given CDAHFD for 12 weeks and then returned to a standard diet or continued a CDAHFD for 4 weeks. (n = 4 per group).
