# Supplementary material for: Estimating Drug Efficacy with a Diet-Induced NASH Model in Chimeric Mice with Humanized Livers
Source: Biomedicines. 2021 Nov 9;9(11):1647. doi: 10.3390/biomedicines9111647 (PMC8615377; doi:10.3390/biomedicines9111647)
Supplement: Supplementary file 1 [file biomedicines-09-01647-s001.zip › Supplementary Table S1.pdf]

Supplementary Table S1. Antibodies used for Immunohistochemistry in this study

Antibody	clone	dilution	Cat No.	Supplier
rat monoclonal anti-F4/80	BM8	200	T-2006	BMA Biomedicals
rat monoclonal anti-Gr1	RB6-8C5	200	550291	BD Pharmingen
mouse monoclonal anti-Ki67	MIB-1	200	M7240	Dako
mouse monoclonal anti-aSMA	1A4	200	A2547	Sigma Aldrich
mouse monoclonal anti-STEM121	-	1000	Y40410	Takara Bio Inc
