# Supplementary material for: Estimating Drug Efficacy with a Diet-Induced NASH Model in Chimeric Mice with Humanized Livers
Source: Biomedicines. 2021 Nov 9;9(11):1647. doi: 10.3390/biomedicines9111647 (PMC8615377; doi:10.3390/biomedicines9111647)
Supplement: Supplementary file 1 [file biomedicines-09-01647-s001.zip › Supplementary Table S2.pdf]

Supplemental Table S2. Primers used for qRT-PCR in this study

gene		sequence	bp
mActa2	F	AGAGGAAGACAGCACAGCCC	20
	R	AGCGTCAGGATCCCTCTCTTG	21
mCcl2	F	ACTCTCACTGAAGCCAGCTC	20
	R	TGAGTAGCAGCAGTGAGTG	20
hCCL2	F	TCTCGCCTCCAGCATGAAAG	20
	R	GGCATTGATTGCATCTGGC	19
mCol1a1	F	TGGCCAAGAAGACATCCCTG	20
	R	CGTGCCATTGTGGCAGATAC	20
mCol1a2	F	TCTTGCTGGCCTACATGGTG	20
	R	CAGGTTGCCCAGATCTACCA	20
mCol3a1	F	TGACTGTCCCACGTAAGCAC	20
	R	GAGGGCCATAGCTGAACTGA	20
hCOL1a1	F	GGCCAAGACGAAGACATCCC	20
	R	ACAGATCACGTCATCGCACA	20
mCOL1a2	F	GCTGTCATTCTACAGGGCTC	20
	R	CATTTGTCTTTTTAGAGCAGCC	22
mCOL3a1	F	GCATACATGGATCAGGCCAG	20
	R	ATTCCCCAGTGTGTTTCGTG	20
mCxcl1	F	TCTCCGTTACTTGGGGACAC	20
	R	CCACACTCAAGAATGGTCGC	20
hCXCL1	F	CTCTACCTGCACACTGTCCTA	21
	R	GCATGTTGCAGGCTCCTCAG	20
mCxcl2	F	TCCAGGTCAGTTAGCCTTGC	20
	R	CGGTCAAAAAGTTTGCCTTG	20
hCXCL2	F	GCTTGTCTCAACCCCGCATC	20
	R	AGCCACCAATAAGCTTCCTCC	21
mHo-1	F	AAGCCGAGAATGCTGAGTTCA	21
	R	GCCGTGTAGATATGGTACAAGGA	23
hHO-1	F	CAACAAAGTGCAAGATTCTGCC	22
	R	TGGCATAAAGCCCTACAGCA	20
mMmp-8	F	CAACATTGCTTTCGTCTCAAGAG	23
	R	GCATGGGCAAGGATTCCATT	20

hMMP-8	F	TGCTCTTACTCCATGTGCAGA	21
	R	TCCAGGTAGTCCTGAACAGTTT	22
mMmp-9	F	GCAGAGGCATACTTGTACCG	20
	R	TGATGTTATGATGGTCCCACTTG	23
hMmp-9	F	GGGACGCAGACATCGTCATC	20
	R	TCGTCATCGTCGAAATGGGC	20
mSOD-1	F	AACCAGTTGTGTTGTCAGGAC	21
	R	CCACCATGTTTCTTAGAGTGAGG	23
hSOD-1	F	GGTGGGCCAAAGGATGAAGAG	21
	R	CCACAAGCCAAACGACTTCC	20
mTimp-1	F	CTTGGTTCCCTGGCGTACTC	20
	R	ACCTGATCCGTCCACAAACAG	21
hTIMP-1	F	CTTCTGCAATTCCGACCTCGT	21
	R	ACGCTGGTATAAGGTGGTCTG	21
mTgf-b1	F	ACGTCACTGGAGTTGTACGG	20
	R	TTTGGGGCTGATCCCGTTG	19
hTGF-B1	F	GGTGGAAACCCACAACGAAATC	22
	R	GGGTTCAGGTACCGCTTCTC	20
mTnf-a	F	ACGTCGTAGCAAACCACCAA	20
	R	ACAAGGTACAACCCATCGGC	20
hTNF-A	F	GCTGCACTTTGGAGTGATCG	20
	R	AAGATGATCTGACTGCCTGGG	21

F: forward primer, R: reverse primer
